# Gene expression signature in adipose tissue of acromegaly patients

**DOI:** 10.1186/1471-2105-15-S10-P28

**Published:** 2014-09-29

**Authors:** Irit Hochberg, Quynh T Tran, Ariel R Barkan, Alan R Saltiel, William F Chandler, Dave Bridges

**Affiliations:** 1 Institute of Endocrinology, Diabetes and Metabolism, Rambam Health Care Campus, Haifa, Israel; 2 Department of Preventive Medicine, University of Tennessee Health Science Center, Memphis, TN 38163, USA; 3 Department of Internal Medicine, University of Michigan, Ann Arbor, MI 48109, USA; 4 Life Sciences Institute, University of Michigan, Ann Arbor, MI 48109, USA; 5 Neurosurgery, University of Michigan, Ann Arbor, MI 48109, USA; 6 Department of Physiology, University of Tennessee Health Science Center, Memphis, TN 38163, USA; 7 Children's Foundation Research Institute, Le Bonheur Children's Hospital, Memphis, TN 38103, USA

## Background

Acromegaly is a rare endocrine disorder with excess growth hormone (GH) production. This disorder has important metabolic effects in insulin resistance and lipolysis. The objective of this study was to explore transcriptional changes induced by GH in adipose tissue.

## Materials and methods

Patients with acromegaly (n=9) or non-functioning pituitary adenoma (n=11) were prospectively observed from March 2011 to June 2012. The patients underwent clinical and metabolic profiling including assessment of HOMA-IR. Explants of adipose tissue were assayed ex-vivo for lipolysis and ceramide levels. Adipose tissue was analyzed by RNA sequencing (RNAseq).

## Results

There was evidence of reduced insulin sensitivity based on the increase in fasting glucose, insulin and HOMA-IR score. We observed several previously reported transcriptional changes (*IGF1*, *IGFBP3*) as well as several novel transcriptional changes, some of which may be important for GH signal regulation (*PTPN3* and *PTPN4*) and the effect of GH on growth and proliferation. Several transcripts could potentially be important in GH-induced metabolic changes. Specifically, induction of *LPL*, *ABHD5*, *ACVR1C* could contribute to enhanced lipolysis and may explain the suggestive enhancement of adipose tissue lipolysis in acromegaly patients as reflected by glycerol release from the explants of the two groups of patients (p=0.09). Higher expression of *SCD* and *TCF7L2* could contribute to insulin resistance. Expression of *HSD11B1* was reduced and *GR* was increased, predicting modified glucocorticoid activity in acromegaly.

## Conclusions

We identified the acromegaly gene expression signature in human adipose tissue. The significance of altered expression of specific transcripts will enhance our understanding of the metabolic and proliferative changes associated with acromegaly.

